# Proteotoxic stresses stimulate dissociation of UBL4A from the tail-anchored protein recognition complex

**DOI:** 10.1042/BCJ20230267

**Published:** 2023-10-11

**Authors:** Takumi Hagiwara, Ryosuke Minami, Chizuru Ushio, Naoto Yokota, Hiroyuki Kawahara

**Affiliations:** 1 Department of Biological Sciences, Tokyo Metropolitan University, Tokyo 192-0397, Japan; 2 Graduate School of Pharmaceutical Sciences, Hokkaido University, Sapporo 060-0812, Japan

**Keywords:** BAG6, polyglutamine disease, proteasome, protein quality control, tail-anchored protein, UBL4A

## Abstract

Inclusion body formation is associated with cytotoxicity in a number of neurodegenerative diseases. However, the molecular basis of the toxicity caused by the accumulation of aggregation-prone proteins remains controversial. In this study, we found that disease-associated inclusions induced by elongated polyglutamine chains disrupt the complex formation of BAG6 with UBL4A, a mammalian homologue of yeast Get5. UBL4A also dissociated from BAG6 in response to proteotoxic stresses such as proteasomal inhibition and mitochondrial depolarization. These findings imply that the cytotoxicity of pathological protein aggregates might be attributed in part to disruption of the BAG6–UBL4A complex that is required for the biogenesis of tail-anchored proteins.

## Introduction

The accumulation of aggregation-prone defective proteins is associated with a wide range of protein-misfolding diseases [1–3]. For example, aberrant folding and fibrillar aggregation by polyglutamine (polyQ) expansion proteins are associated with cytotoxicity in several neurodegenerative disorders, such as Huntington's disease (HD), Machado–Joseph disease (MJD), and dentatorubral-pallidoluysian atrophy (DRPLA) [4–7]. A minimum polyQ tract length of 30 to 40 glutamines is necessary to cause these diseases, and polyQ repeats above the threshold cause the proteins to adopt a non-native conformation that is highly prone to self-associate into high molecular weight, ubiquitin-positive aggregates [7]. Inclusion body formation has been hypothesized to contribute to disease pathogenesis through the indirect inhibition of the ubiquitin/proteasome-dependent pathway [8–13]. Several investigations have also suggested that expanded polyQ stretches contribute to the development of neuronal degeneration by abrogating several synaptic vesicle proteins that are essential for membrane trafficking, axonal transport, and exocytotic transmitter release [14–19]. However, because the defects observed to be induced by polyQ moieties are extremely diverse, a co-ordinated understanding of the pathological basis of polyQ toxicity has been limited.

The tail-anchored protein is a family of C-terminally anchored transmembrane domain proteins that includes the ubiquitin ligases required for endoplasmic reticulum-associated degradation (ERAD) and a large member of soluble N-ethylmaleimide-sensitive factor attachment receptor (SNARE) components [20–25]. Because the members of the tail-anchored protein family do not possess a signal sequence at the N-terminus, they must be inserted into the endoplasmic reticulum (ER) membrane via post-translational pathways [24,25], which is a distinct mechanism to that of most transmembrane proteins that are integrated mainly by the signal sequence-dependent co-translational process [26]. In yeast, the GET (Guided Entry of Tail-anchored proteins) pathway plays a critical role in tail-anchored protein biogenesis [27–30]. In the first step of this pathway, the chaperone-like protein Sgt2 captures the C-terminal transmembrane domain of tail-anchored proteins. Subsequently, Get5 and Get4 facilitate complex formation of Get3-tail-anchored protein by tethering Sgt2 to Get3 [24,31–34]. Tail-anchored proteins are then transferred from Get3 to the ER-resident translocon-like proteins Get1/Get2 and finally inserted into the ER membrane [25,28,30].

**Figure 1.**
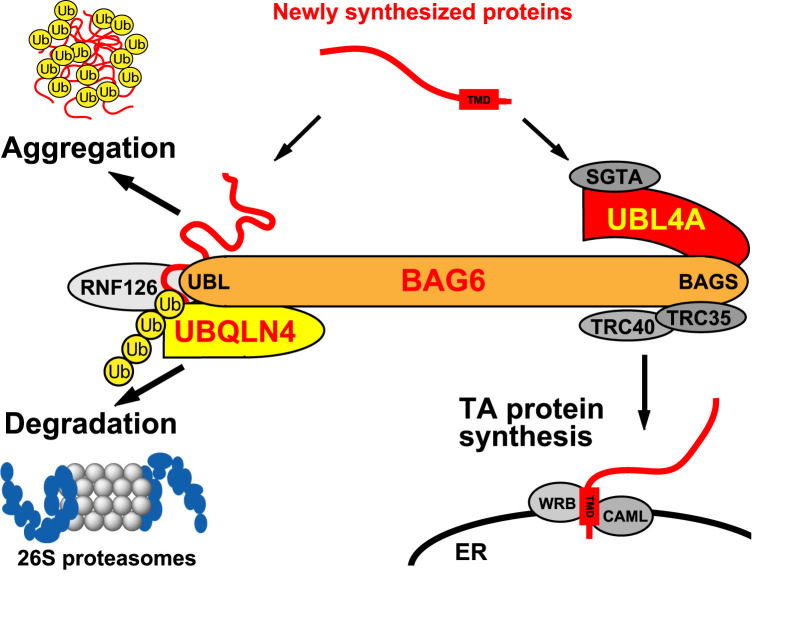
Schematic of the functional duality of BAG6 complex. UBL4A, TRC35, and TRC40, mammalian homologues of yeast Get5, Get4, and Get3, respectively, are complexed with the C-terminus of BAG6 for the correct insertion of tail-anchored (TA) proteins into the lipid bilayer of the ER membrane (TA protein synthesis). In contrast, the N-terminal region of BAG6 is complexed with the ubiquitin ligase RNF126 [82] and UBQLN4, an ubiquitin receptor for 26S proteasomal targeting, and both of these are critical for the degradation of newly synthesized defective proteins. It has been reported that BAG6 tends to be enfolded into the aggresomes under proteasomal inhibition with polyubiquitinated aggregation-prone polypeptides. TMD, transmembrane domain; ER, endoplasmic reticulum.

Mammalian homologues of GET components are also responsible for tail-anchored protein biogenesis (Figure 1) [35–40]. TRC40 and TRC35, the components of the transmembrane domain recognition complex (TRC), are the mammalian homologues of yeast Get3 and Get4, respectively [35,41], and facilitate transfer of the substrates to Get1 (also known as WRB) and CAML, the ER-resident translocation machinery for mammalian tail-anchored proteins (Figure 1) [42]. UBL4A, the mammalian homologue of yeast Get5, and the C-terminal region of BAG6 are also necessary for connecting TRC40/Get3 and tail-anchored proteins for their correct insertion into the lipid bilayer of the ER membrane (Figure 1) [35,37,41,43]. Should assembly fail, mislocalized tail-anchored proteins are sent to the ubiquitin-mediated degradation pathway via BAG6 and SGTA [44–51]. Thus, a series of studies suggests that the BAG6–UBL4A complex captures and shields the hydrophobic transmembrane domain of tail-anchored proteins after their ribosomal release and determines whether they are assembled into the ER membrane or degraded in the cytoplasm (Figure 1) [30,35,39,44,45,52,53].

BAG6 has been identified as an essential factor in the ubiquitin-mediated degradation of newly synthesized defective polypeptides [39,44,45,52,54–57]. It has also been reported that the N-terminal region of BAG6 is critical for the surveillance of aberrant polypeptides with a hydrophobic tail that are derived from the translation of noncoding regions [58]. BAG6 physically interacts with polyubiquitinated aggregation-prone polypeptides (Figure 1), and BAG6 indeed colocalizes with ubiquitin-positive cytoplasmic inclusions such as aggresomes [54] and polyQ inclusion bodies [59]. Because the BAG6–UBL4A complex is also involved in tail-anchored protein biogenesis, this complex seems to possess a dual function in defective protein degradation and tail-anchored protein biogenesis (Figure 1) [39,52]. However, the functional relationship between UBL4A and defective protein aggregation remains elusive.

In this study, we found that disease-associated inclusions induced by DRPLA-derived elongated polyQ chains or proteasome inhibitor-induced aggresome formation disrupt the association between UBL4A and BAG6. Since the UBL4A–BAG6 complex is an essential mediator of tail-anchored protein biogenesis [35,39,41,52], these findings led us to propose a hypothesis in which the cytotoxicity of pathological polyQ aggregation might partly be due to disruption of this complex. Using a NanoBiT split luciferase system, we showed that UBL4A dissociates from the BAG6 complex in response to not only polyQ aggregation but also mitochondrial depolarization. Therefore, the BAG6–UBL4A complex might be a candidate mediator of the wide variety of pathological processes associated with proteotoxic stresses.

## Results

### UBL4A-free species of BAG6 are predominantly enfolded into insoluble aggregates

**Figure 2.**
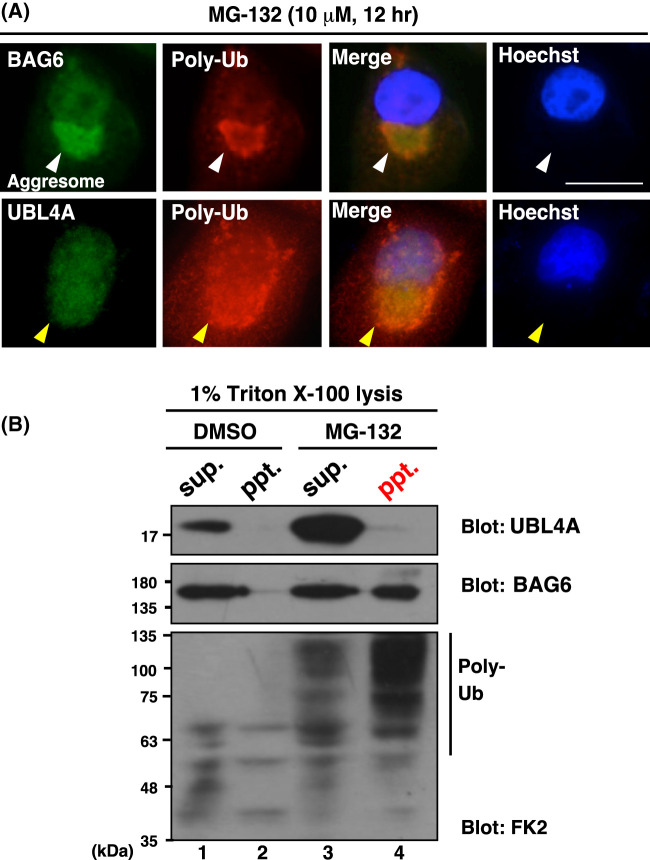
UBL4A is absent from the MG-132-induced insoluble aggresome fraction. (**A**) Immunosignals of BAG6 (green in upper panels) and polyubiquitin (red) were positive in insoluble aggresomes following MG-132 treatment, while those of UBL4A (green in lower panels) were negative. HeLa cells were treated with MG-132 (10 µM) for 12 h, and fixed cells were stained with anti-BAG6, anti-UBL4A, and anti-polyubiquitin antibodies. The positions of the aggresomes are indicated by arrowheads. Hoechst nuclear stains are merged in right panels. Bar: 10 µm. (**B**) FLAG-UBL4A-transfected HeLa cells were treated with 10 µM MG-132 or DMSO (as a negative control) for 6 h. Cellular homogenates with 1% Triton X-100 lysis buffer were fractionated by centrifugation at 20 000×***g*** to obtain the supernatant (sup.) and pellet (ppt.) fractions, which were immunoblotted with anti-FLAG (UBL4A), anti-BAG6, and anti-polyubiquitin (FK2) antibodies.

BAG6 has been reported to possess an affinity for aggregation-prone defective polypeptides [39,44,45,52,54]. Consequently, BAG6 tends to be enfolded into insoluble protein aggregates that are rich in polyubiquitinated defective proteins [54]. Indeed, immunocytochemical signals for BAG6 were strongly positive in the ubiquitin-positive perinuclear inclusions known as aggresomes [60] in proteasome-inhibited HeLa cells (Figure 2A, indicated by white arrowheads).

Because BAG6 is tightly complexed with UBL4A/Get5 [61,62], a known BAG6 partner for tail-anchored protein biogenesis [35,41], we examined whether UBL4A is also included in MG-132-induced aggresomes. Contrary to our expectations, the immunocytochemical signals of UBL4A were essentially negative for aggresome stain (Figure 2A, yellow arrowheads).

Cell fractionation analysis supported this finding. As shown in Figure 2B, MG-132-induced ubiquitin-positive insoluble aggregates scarcely contained UBL4A (Figure 2B, lane 4, UBL4A blot) but were rich in BAG6 and insoluble polyubiquitinated proteins (Figure 2B, lane 4, BAG6 and poly-Ub blots). These observations suggest that a pool of BAG6 species that is free from UBL4A association is selectively enfolded into the insoluble fraction. Alternatively, UBL4A might dissociate from BAG6 in proteasome-inhibited cells. In either case, UBL4A-free species of BAG6 predominantly associated with aggregation-prone defective polypeptides.

### UBL4A dissociates from BAG6 complex in response to proteotoxic stresses

**Figure 3.**
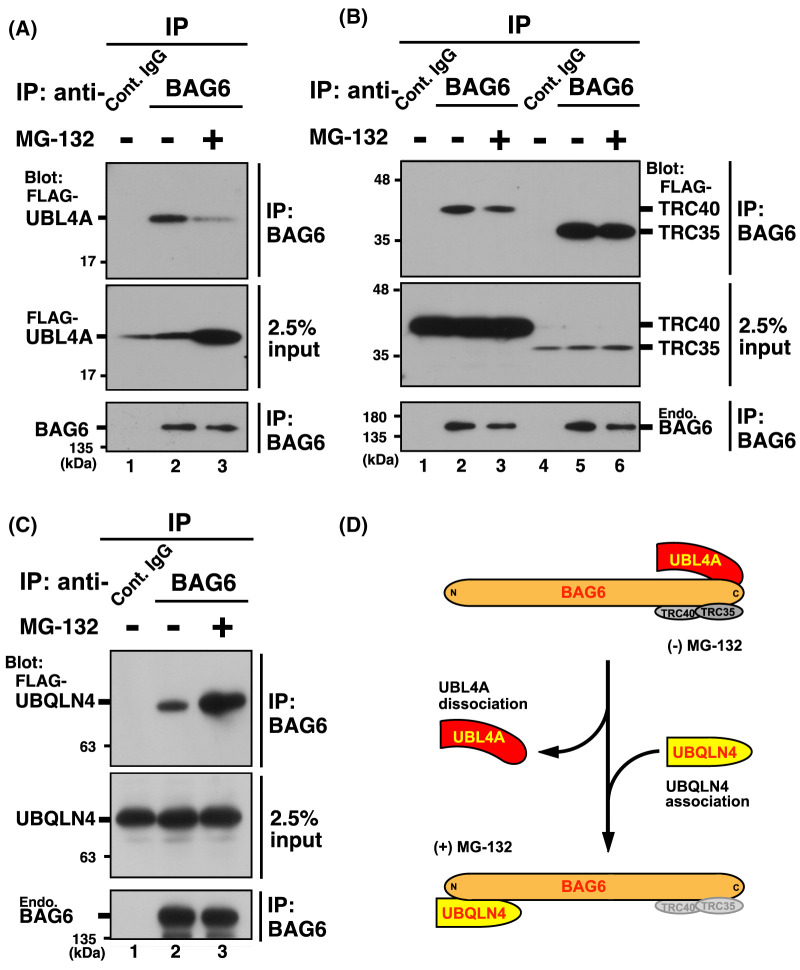
Association of UBL4A and BAG6 is suppressed by proteasome inhibition in HeLa cells. (**A** and **B**) MG-132 treatments reduced the amounts of BAG6-associated FLAG-UBL4A in soluble fractions of HeLa cells. Expression plasmids of FLAG-tagged UBL4A, TRC35, and TRC40 were transfected into HeLa cells. After 24 h of transfection, the cells were treated with (+) or without (−) 10 µM MG-132 for 4 h. Anti-BAG6 antibody co-precipitated UBL4A with endogenous BAG6 less efficiently in the presence of MG-132 (**A**, top panel), while the co-precipitation of TRC35 and TRC40 with BAG6 was not influenced by this treatment (**B**, top panel). Note that MG-132 treatment resulted in the accumulation of total soluble UBL4A protein, probably due to its stabilization (**A**, second panel). (**C**) MG-132-treatment of HeLa cells stimulated the association of UBQLN4 with BAG6. After 24 h of FLAG-UBQLN4 transfection, the cells were treated with (+) or without (−) 10 µM MG-132 for 4 h. Anti-BAG6 antibody co-precipitated UBQLN4 with endogenous BAG6 more efficiently in the presence of MG-132 (**C**, top panel), while the expression level of UBQLN4 was not affected by this treatment (**C**, middle panel). (**D**) Schematic image of mutually exclusive associations of UBQLN4 and UBL4A with BAG6 in cells treated with (+) or without (−) proteasome inhibitor MG-132.

Previous surface plasmon resonance study suggested that the direct interaction between BAG6 and UBL4A is remarkably strong (with an *in vitro* dissociation constant calculated to be 2.2 nM) [62]. Considering the relatively high expression levels of endogenous BAG6 and UBL4A proteins in living cells (a 720 nM protein concentration for BAG6 and 700 nM for UBL4A in HEK293T cells according to the estimation in OpenCell; https://opencell.czbiohub.org), we suspected that the dissociation of UBL4A from BAG6 would be unlikely. Contrary to this expectation, we found that blockage of the proteasome greatly reduced the amount of UBL4A that was co-immunoprecipitated with soluble BAG6 (Figure 3A, upper panel, compare lanes 2 and 3), even though the BAG6-free form of UBL4A, which has been shown to have a short half-life, apparently stabilized and accumulated in the soluble fraction of MG-132-treated cells (Figure 3A, middle panel, compare lanes 2 and 3). The quantity of other BAG6-associated TRC proteins, TRC35 and TRC40, was unaltered in BAG6 precipitate under identical conditions (Figure 3B, upper panel). These observations imply that only a minor portion of UBL4A is associated with the BAG6 complex in proteasome-suppressed cells. It is interesting to note that BAG6 binding to UBQLN4, a ubiquitin receptor protein required for the degradation of defective transmembrane domain proteins, was greatly augmented by MG-132 treatment (Figure 3C, upper panel), as reported previously [56]. These findings suggested that two distinct BAG6-associated proteins, UBL4A and UBQLN4, showed opposing sensitivities to proteasomal inhibition for complex formation with BAG6 (Figure 3D). Collectively, the association of UBL4A with the BAG6 complex in living cells could be changed dynamically in response to proteotoxic stresses such as proteasome impairment and/or aggregate formation.

### Forced expression of UBL4A blocks BAG6 translocation to the insoluble fraction

**Figure 4.**
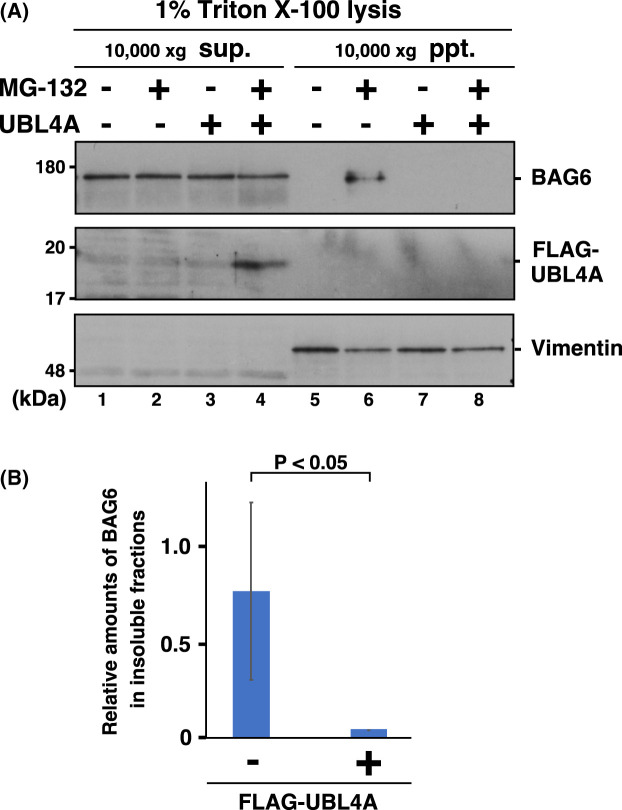
Forced expression of UBL4A blocks the translocation of BAG6 to the insoluble fractions. (**A**) Translocation of endogenous BAG6 to insoluble aggregates was suppressed by overexpression of UBL4A in proteasome-suppressed cells. After cells were treated with 5 µM MG-132 for 18 h, the cells were fractionated to soluble (sup.) and insoluble (ppt.) fractions by centrifugation at 10 000×***g*** with buffer containing 1% Triton X-100. The resulting sup. and ppt. fractions were subjected to western blot analyses with anti-BAG6 and anti-FLAG (UBL4A) antibodies. Vimentin was used as a loading control and insoluble fraction marker. Note that increased amount of FLAG-UBL4A can be detected in the soluble fraction of MG-132-treated cells (lane 4), even though it was essentially negative in insoluble fractions (lane 8). (**B**) The graph indicates the quantified signal intensities of the endogenous BAG6 protein in insoluble fractions relative to the anti-vimentin signals. Experiments involved three biologically independent replicates to compute statistical significance. Data are presented as means ± standard deviation (S.D.) and were analyzed using Student's *t*-test. A *P*-value <0.05 was considered statistically significant.

Although BAG6 was shown to be critical for preventing protein aggregate formation [54], the role of UBL4A in protein quality control has not been addressed adequately. Therefore, we investigated whether ectopically overexpressed UBL4A had any effects on protein aggregation events in MG-132-treated cells. As reported previously [54], MG-132 treatment stimulated BAG6 translocation to the insoluble fraction (Figure 4A, upper panel, compare lanes 5 and 6). Our finding was that forced expression of FLAG-tagged UBL4A prevented BAG6 translocation to the insoluble fraction (Figure 4A, upper panel, compare lanes 6 and 8). Quantification of the amount of BAG6 in the insoluble fractions suggested significantly fewer BAG6-positive inclusions in UBL4A-expressing cells (Figure 4B). These observations suggest a role for UBL4A in the prevention of BAG6 incorporation into proteotoxicity-stimulated inclusions. Alternatively, excess expression of UBL4A might suppress aggregate formation in living cells.

### Aggregation-prone polyQ protein disrupts the UBL4A–BAG6 complex

It has been reported that proteins with a long polyglutamine chain tend to form aggresome-like protein inclusions even in the absence of proteasome inhibitor [6,63,64]. Because the expression of polyQ-expanded atrophin-1 is associated with the formation of perinuclear inclusions [6,64], we examined whether UBL4A dissociates from the BAG6 complex in response to polyQ aggregation. We expressed polyQ-green fluorescent protein (GFP) fusion proteins with polyQ tracts of normal (32 residues) or pathological (79 residues) lengths (designated Q32-GFP and Q79-GFP, respectively) fused to the polyQ flanking sequence derived from human atrophin-1 [5,63,65]. Although the Q32-GFP fusion protein exhibited a diffuse GFP localization pattern throughout the cells, the Q79-GFP fusion protein formed discrete nuclear/perinuclear aggregates (Supplementary Figure S1) [64].

**Figure 5.**
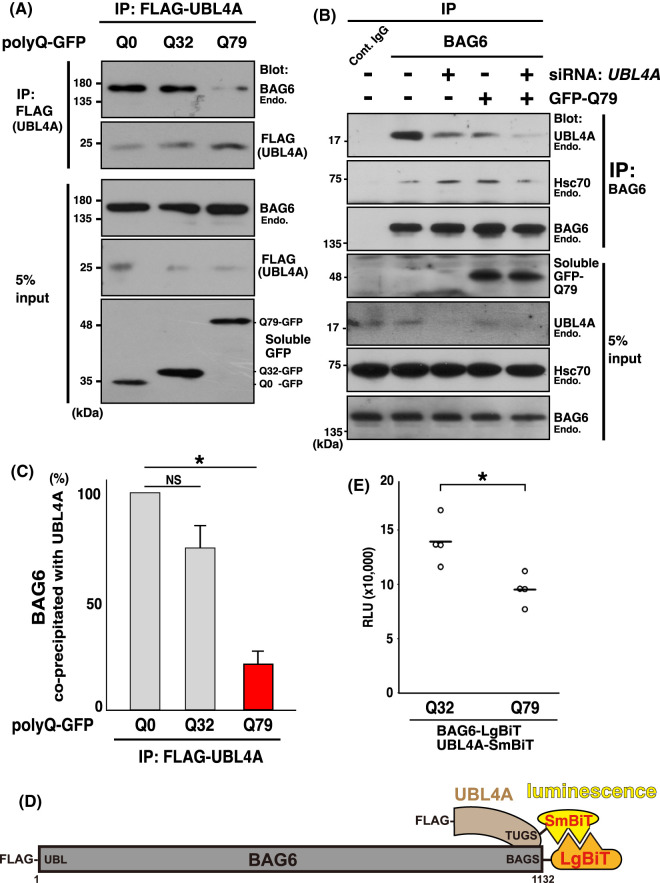
Aggregation of expanded polyQ protein disrupts of the UBL4A–BAG6 association. (**A**) FLAG-UBL4A was immunoprecipitated from polyQ-GFP-expressing HeLa cell extracts (5% input), and precipitates (IP: FLAG) were subjected to western blot analysis with an anti-BAG6 antibody to quantify the amount of endogenous BAG6 that was associated with FLAG-UBL4A (top panel). Expressions of polyQ proteins were verified by GFP blots (bottom panel). (**B**) Endogenous BAG6 was immunoprecipitated by anti-BAG6 (or non-immune control IgG) from the cell extracts of Q79-GFP (+) or mock (−)-expressing HeLa cells. Precipitates were subjected to western blot analysis with an anti-UBL4A antibody to quantify the amounts of BAG6-associated UBL4A protein (top panel). siRNA for *UBL4A* was performed (+) to confirm the validity of the UBL4A immunosignal. Anti-BAG6 and anti-Hsc70 blot verified the quantitative immunoprecipitation. The Hsc70 blot in the input panel shows the loading control. ‘Endo.' stands for endogenous proteins. (**C**) Quantitative evaluation of the endogenous BAG6 signal that co-precipitated with FLAG-UBL4A from polyQ-GFP-expressing cell extracts. Note that the value of co-precipitated BAG6 from cells not expressing polyQ protein (Q0) was used as the standard (100%). Experiments involved six biologically independent replicates to compute statistical significance. Data are presented as means ± standard deviation (S.D.) and were analyzed using Student's *t*-test. A *P*-value <0.05 was considered statistically significant. (**D**) Schematic of the NanoBiT split luciferase assay used in this study. Large-BiT (LgBiT, 17.6 kDa) and Small-BiT (SmBiT, 11 amino acids long) tags were fused to the C-termini of BAG6 and UBL4A, respectively, to detect the binding efficiency of these proteins in cells. Note that complex formation between BAG6–UBL4A is directly mediated through their C-terminal BAGS-TUGS domains [61,62]. The numbers denote the corresponding amino acid positions of full-length BAG6 protein. (**E**) PolyQ32 and polyQ79 proteins were expressed in HeLa cells with BAG6-LgBiT and UBL4A-SmBiT. Forty-eight hours after transfection of expression plasmids, NanoBiT-derived luminescence were measured, and the respective values normalized by cell number (RLU) were plotted on a graph. Welch's *t*-test *n* = 4. * *P*-value <0.05 was considered statistically significant.

We found that the expression of aggregation-prone polyQ protein modulated the association between UBL4A and BAG6 (Figure 5). Indeed, when protein aggregates were induced by expression of the expanded polyQ protein (Q79-GFP) in HeLa cells, the amount of endogenous BAG6 that co-precipitated with FLAG-UBL4A greatly decreased compared with the short polyQ protein (Q32-GFP) (Figure 5A). Reciprocally, the average amounts of endogenous UBL4A protein that co-precipitated with endogenous BAG6 from Q79-GFP cell extracts significantly decreased compared with those from Q32-GFP-expressing extracts (Figure 5B). We confirmed that the 17 kDa signal that co-precipitated with FLAG-BAG6 was specific to endogenous UBL4A because siRNA-mediated knockdown of UBL4A effectively reduced the intensities of the corresponding signals (Figure 5B). Such a reduction in the BAG6–UBL4A complex was specifically induced by expanded polyQ tracts, since forced expression of non-pathological Q32-GFP under identical conditions showed only moderate effects on the UBL4A–BAG6 complex (Figure 5C).

### NanoBiT-based luminescence assay supports polyglutamine aggregation-induced dissociation of BAG6–UBL4A complex

To further support the dissociation of the BAG6–UBL4A complex with polyQ aggregation in living cells, we took advantage of the NanoBiT split luciferase system. In this assay, the C-termini of BAG6 and UBL4A were fused to large BiT (LgBiT) and small BiT (SmBiT) tag sequences, respectively (Figure 5D), and these fusion proteins were co-expressed in HeLa cells. Because the C-termini of BAG6 and UBL4A, BAGS domain and TUGS domain, respectively, are critical regions for BAG6–UBL4A association (Figure 5D) [62], complex formation between BAG6 and UBL4A in cells should position LgBiT and SmBiT close to each other (Figure 5D), thereby reconstituting the full enzymatic activity of luciferase. In accordance with this idea, we detected very strong luminescence signals in cells co-expressing BAG6-LgBiT and UBL4A-SmBiT (Supplementary Figure S2A). Such luciferase activity was completely lost when UBL4A-SmBiT was replaced with SmBiT control vector (Supplementary Figure S2A). Importantly, luciferase activity was largely abrogated by point mutations in the BAGS domain of BAG6 (V1068R and L1086R mutants) [62], supporting the specificity of the BAG6–UBL4A interaction in this assay system (Supplementary Figure S2B). With this NanoBiT split luciferase system, we confirmed that the expression of Q79-GFP resulted in a significant reduction in the BAG6–UBL4A interaction compared with the case for Q32-GFP expression (Figure 5E). These observations together suggest that aggregation-prone polyQ moieties disrupt BAG6–UBL4A complex formation and thereby let BAG6 separate from UBL4A.

### UBL4A dissociates from BAG6 complex in response to mitochondrial stresses

**Figure 6.**
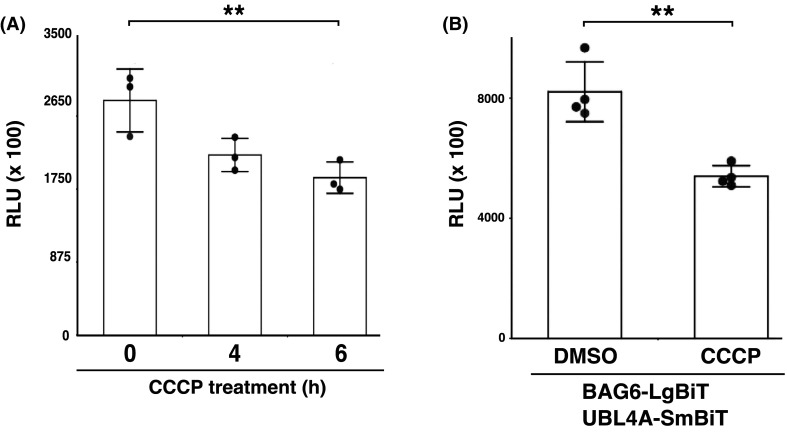
Mitochondrial depolarization leads to dissociation of the UBL4A–BAG6 complex. (**A**) Time-dependent dissociation of BAG6–UBL4A complex by treatment with CCCP, a mitochondrial uncoupling agent. Twenty-four hours after transfection with BAG6-LgBiT and UBL4A-SmBiT expression vectors, FLAG-Parkin-expressing HeLa cells were treated with 20 µM CCCP for the indicated time periods, and NanoBiT-derived luminescence was measured, and the respective values normalized by cell number (RLU) were plotted on a graph. Student's *t*-test, *n* = 3. ** *P* < 0.01. (**B**) Twenty-four hours after transfection with BAG6-LgBiT and UBL4A-SmBiT expression vectors, FLAG-Parkin-expressing HeLa cells were treated with 20 µM CCCP for 4 h. Four-hour treatment with dimethyl sulfoxide (DMSO, a solvent for CCCP) was used as a negative control. Welch's *t*-test, *n* = 4. ** *P* < 0.01.

Using the NanoBiT split luciferase system, we examined the effect of various cellular stresses on the BAG6–UBL4A complex. Although ER stress induced by tunicamycin did not affect NanoBiT-derived luminescence (Supplementary Figure S3A), we found that mitochondrial decoupling mediated by carbonyl cyanide m-chlorophenyl hydrazone (CCCP), an inhibitor of mitochondrial oxidative phosphorylation that induces mitochondria damage [66,67], evoked a decrease in NanoBiT split luciferase activity (Figure 6). Since CCCP-induced clustering of mitochondria is Parkin-dependent, we used cells stably expressing Parkin. CCCP treatment for 4 h was enough to stimulate the dissociation of BAG6 and UBL4A (Figure 6A). Biologically independent replicates support a significant difference in luminescence intensity between CCCP and DMSO control treatments (Figure 6B). Luminescence signal intensity derived from LgBiT-PRKAR2A and SmBiT-PRKACA (these are provided by the manufacturer as a positive control pair) were not significantly reduced by CCCP (Supplementary Figure S3B), suggesting that mitochondrial depolarization did not itself affect luciferase activity. These results collectively suggest that the BAG6–UBL4A complex might be linked not only to protein aggregation events, but also to the CCCP-induced mitochondrial depolarization stress response.

## Discussion

PolyQ aggregation is known to be central to the pathology of neurological diseases [1,7], although the molecular basis of the polyQ toxicity remains controversial. PolyQ aggregations have been suggested to cause various dysfunctions, such as impairment of the ubiquitin–proteasome system and defects in membrane vesicle fusion. For example, Huntington's disease model mice exhibit decreased cell surface expression of channel proteins, which are essential for presynaptic neurotransmitter release [68]. Chhetri et al. [19] recently reported that the expression of mutant huntingtin with long polyQ tracts (HTT Q111) interfered with the endosomal vesicular trafficking and cell surface expression of membrane-bound receptors and transporters [19,69]. These reports imply that alterations in vesicular trafficking in neurodegenerative disorders are caused by polyglutamine expansion. However, it remains unclear how polyQ tracts affect the endosomal trafficking and recycling of membrane proteins to the cell surface.

**Figure 7.**
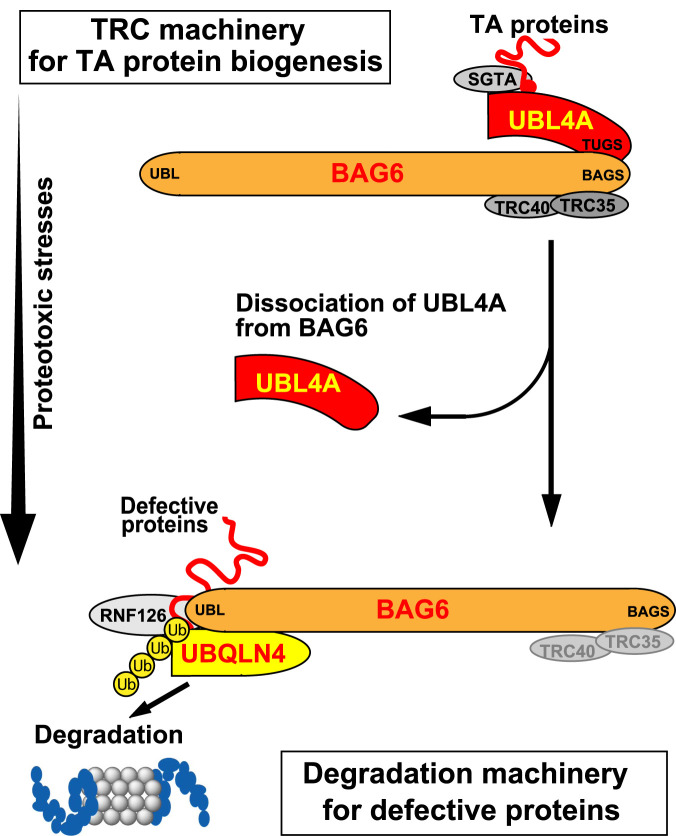
Possible model for proteotoxic stress-induced defects in TRC machinery. TRC machinery including BAG6–UBL4A complex participates in tail-anchored (TA) protein biogenesis. Once proteotoxic stresses (such as polyQ aggregation and mitochondrial depolarization) are induced, the BAG6–UBL4A complex dissociates, letting BAG6 separate from UBL4A. Because the UBL4A–BAG6 complex is a core of mammalian TRC machinery, its dissociation may result in the attenuation of tail-anchored protein synthesis, thereby leading to partial cytotoxicity. Note that many of these components are likely to form a dimer (or multimer), although they are described here as a monomer for simplicity.

Our finding in this study is that the expression of aggregation-prone polyQ protein in mammalian cells abolished BAG6–UBL4A complex formation (Figure 5). Proteasome inhibition also reduced the UBL4A-associated form of BAG6 in soluble cell extracts (Figure 3). UBL4A was originally identified as an X-chromosome-encoded ubiquitin-like domain protein [70], which was later shown to be a homologue of yeast Get5 [35,41], an essential component of the tail-anchored protein recognition complex in yeast. Tail-anchored proteins are linked to various aspects of vesicular trafficking [20], ERAD [22,23], and proteasomal assembly [71]. For example, many of the components of intracellular vesicular trafficking machinery, such as t-SNARE syntaxins and v-SNARE synaptobrevins/VAMP, belong to a typical family of tail-anchored proteins [25], and SNARE proteins are sensitive to disruption of GET pathway components [42,72,73]. The mammalian tail-anchored protein recognition complex (comprising BAG6, TRC35, and UBL4A) also plays a role in the efficient assembly of tail-anchored proteins [35,41,61], and disruption of the BAG6–UBL4A complex may have a critical impact on the synthesis of tail-anchored proteins necessary for vesicular trafficking events. In accordance with this notion, proper assembly of the SNARE family tail-anchored proteins into the organelle membranes was disturbed under depletion of TRC components [73–75]. BAG6 is also known as a regulator of membrane trafficking in mammalian cells [74,76,77]. Therefore, defective endosomal trafficking induced by long polyQ tract accumulations may be partly explained by defective BAG6–UBL4A complex formation. Other neurodegenerative diseases that are caused by the formation of protein aggregates should also result in disruption of the BAG6–UBL4A complex. In agreement with this idea, aggregation-prone TDP-43 species could be polyubiquitinated by a BAG6 complex containing TRC35, but lacking UBL4A [78], suggesting that commitment of BAG6 to protein quality control could be accomplished by disengagement with UBL4A. BAG6 is also involved in the core particle assembly of 20S proteasome complex through the TRC/GET pathway [71]. Our hypothesis is that the cytotoxicity of pathological protein aggregation (including polyQ) is attributable in part to disruption of the essential functions of the BAG6–UBL4A complex (Figure 7). Such a possibility should be further examined in future study.

Considering the extremely high affinity (*K*_d_ ∼ 10^−9^ M) of the *in vitro* interaction between BAG6 and UBL4A [62], the dissociation of the BAG6 complex by polyQ aggregation in cells was unexpected. This study suggests that UBL4A–BAG6 complex formation is dynamic in nature and that complex dissociation occurs under conditions of proteotoxic stresses (Figure 7). As such, BAG6–UBL4A complex may provide a typical example for stress-sensitive molecular triage, which determines the fate of newly synthesized tail-anchored proteins for ER membrane targeting or ubiquitin-mediated degradation [79]. In the case of stress induction, the UBL4A cycle might stay retarded at the dissociation state, leaving BAG6 free from UBL4A, and enable BAG6 to facilitate aberrant protein destruction. It could be that there is a need to prioritize the elimination of defective proteins over the synthesis of tail-anchored proteins under conditions with an accumulation of defective proteins. This may explain why the predominant BAG6 complex is the one without UBL4A in these circumstances (Figure 7). Such novel regulation of the BAG6–UBL4A complex might play an important role in protein metabolism in stressed cells, especially in modulating the progression of neurodegenerative diseases caused by the accumulation of expanded polyQ and related aggregation-prone proteins. Elucidation of the mechanism underlying how the aggregation-prone proteins modulate the BAG6–UBL4A complex in the regulation of neurodegeneration is likely to provide important insights into basic pathomechanistic principles for a number of protein-misfolding diseases. Therefore, this should be extensively exploited for therapeutic purposes in a future study.

## Materials and methods

### Constructs

Full-length cDNA for human *BAG6* and *UBL4A* was amplified by PCR from HeLa cDNA libraries. The PCR products were subcloned into the pCI-neo-based mammalian expression vector (Promega) with appropriate tags. Truncated and mutated versions of *BAG6* and *UBL4A* genes were prepared by PCR with pCI-neo-based vectors as templates. Vectors were used for experiments after verification of the sequence of inserted DNA. To express polyQ-GFP proteins in mammalian cell lines, *Nhe*I-*Not*I fragments of pCKX2004 vector and pCKX2001 vector (kindly gifted by Dr. K. Yamanaka) were excised and cloned into *Nhe*I and *Not*I-digested pCI-neo-3FLAG vector. These expression plasmids contain DRPLA-derived polyQ stretches fused to the GFP gene [63]. The GFP gene is derived from pEGFP-N1 vector (Clontech).

### Mammalian cell culture and transfection

HeLa cells were cultured in Dulbecco's modified Eagle's medium (Sigma) supplemented with 10% heat-inactivated fetal calf serum at 37°C under a 5% CO_2_ atmosphere. DNA transfection was performed using Lipofectamine 2000 (Invitrogen) following the supplier's instructions. For the expression of polyQ proteins, HeLa cells were transfected with expression vectors (encoding DRPLA-derived polyQ79 or polyQ32 stretches fused to the GFP) using PEI MAX (Cat# 24765-1, Polysciences, Inc), and harvested at 48 h after transfection for further analyses.

### Small interfering RNA oligonucleotides

For knockdown analysis of UBL4A, the oligonucleotide specifically covering the sequence 5′-TCTGGCAGCTGATCTCCAA-3′ was synthesized (SIGMA Genosys) and used for transfection with Lipofectamine 2000 according to the protocol provided by the manufacturer. The oligonucleotide covering the sequence of luciferase described in Buscà et al. [80] was used as a negative control. The efficacies of each siRNA were verified by immunoblot with anti-UBL4A antibody.

### Immunoprecipitation and western blotting

HeLa cells were washed twice with ice-cold phosphate-buffered saline and lysed with Buffer A (20 mM Tris–HCl pH7.5, 5 mM EDTA, 150 mM NaCl, 5% glycerol, and 1% Nonidet P-40). The lysates were sonicated for 1 s and centrifuged at 20 000×***g*** for 20 min at 4°C, and the resulting supernatant was incubated with anti-BAG6 antibody stabilized to Protein A Sepharose (GE Healthcare) for 2 h at 4°C. After the beads were washed four times with cold Buffer A, the purified complexes were eluted with SDS sample buffer.

For western blotting, the whole cell lysates and the immunoprecipitated materials were separated by SDS–PAGE and transferred onto nitrocellulose membranes (Bio-Rad).

The membranes were immunoblotted with specific antibodies as indicated and then incubated with horseradish peroxidase-conjugated antibody against mouse or rabbit immunoglobulin (Amersham Life Science, Buckinghamshire, U.K.), followed by detection with ECL Western blotting detection reagents (Amersham Biosciences). The rabbit polyclonal antibody against BAG6/Scythe was obtained as described previously [54]. To prepare antiserum against UBL4A, rabbits were immunized with recombinant full-length UBL4A. Alternatively, commercially available anti-UBL4A antibody was purchased (Cat. No. 14253-1-AP, Proteintech). The following antibodies were purchased and used for immunological analyses; anti-polyubiquitin FK2 (MBL or Nippon Bio-Test Laboratories Inc.), anti-Hsp70/Hsc70 (MBL), anti-Vimentin (Santa Cruz). anti-Flag M2 monoclonal (Sigma), and anti-GFP (Roche).

### Cell fractionation assay

HeLa cells were transfected with a FLAG-UBL4A expression vector. After 24 h of transfection, the cells were treated with 10 µM MG-132 or DMSO (as a negative control) for 6 h and harvested with lysis buffer (20 mM Tris–HCl pH 7.5, 1 mM EDTA, 150 mM NaCl, 5% glycerol, 1% Triton X-100). After sonication and centrifugation at 10 000×***g***, insoluble pellets were extensively washed with lysis buffer. Both the soluble (sup.) and insoluble (ppt.) fractions obtained were boiled with SDS sample buffer and subjected to western blot analysis with anti-FLAG, anti-BAG6, and anti-polyubiquitin (FK2) antibodies. Note that the dilution ratios of the insoluble fractions by SDS sample buffer for electrophoresis were adjusted to that of the corresponding soluble fractions.

### Microscopy

Cells were grown on micro cover glass (Matsunami), fixed in 4% paraformaldehyde, permeabilized by 0.1% Triton X-100, and blocked with 3% bovine serum albumin in PBS. Incubation with a series of primary antibody was conducted at room temperature for 1 h and was followed by 1 h incubation at room temperature with secondary antibodies. For secondary antibodies, Alexa^TM^ 594-conjugated and Alexa^TM^ 488-conjugated anti-rabbit or -mouse IgG antibodies (Molecular Probes) were used at 1 : 800 dilutions. To observe the nucleus, cells were stained with 2.5 µg/ml diamidino-2-phenylindole (DAPI) or Hoechst 33342 in PBS at the time of antibody staining. Immunofluorescent images were obtained with a BIOREVO BZ9000 fluorescence microscope (Keyence) and an LSM510 inverted confocal microscopy system (Carl Zeiss).

### Nanobit split luciferase assay

pBiT vectors for the NanoBiT split luciferase assay were purchased from Promega (NanoBiT PPI MCS starter systems, Cat No. N2014). The C-terminus of full-length BAG6 protein was fused to the LgBiT tag (158 amino acids long, encoded in pBiT1.1-C vector, Cat No. N196A, Promega) with a glycine linker (GSSGGGGSGGGGSSG), and the C-terminus of full-length UBL4A protein was fused to the SmBiT tag (11 amino acids long, encoded in pBiT2.1-C vector, Cat No. N197A, Promega) with a glycine linker. To confirm the expression of the proteins, the N-termini of BAG6 and UBL4A were fused to 3xFLAG tag. NanoBiT® Negative Control Vector (Cat. No. N202A, Promega) was used as a negative control. LgBiT-PRKAR2A (Cat. No. N203A, Promega) and SmBiT-PRKACA (Cat. No. N204A, Promega) control vectors were used as positive controls.

Primer sequences for amplification of cDNA encoding the full-length BAG6 protein were

5′-CCAGATCTATGGAGCCTAATGATAGTACCAGTACC-3′

5′-TTCTCGAGCCAGGATCATCAGCAAAGGCCCGCTGG-3′

for subcloning into the pBiT 1.1-C vector.

Primer sequences for amplification of cDNA encoding the full-length UBL4A protein were

5′-GGAGATCTATGCAGCTGACGGTGAAGGCGCTGCAG-3′

5′-CCCTCGAGCCTTTGGAGAAGCCCTTCTCCATTGTC-3′

for subcloning into the pBiT 2.1-C vector.

HeLa cells were co-transfected with pBiT 1.1 vector, pBiT 2.1 vector, and polyglutamine expression vectors (Q78 or Q34) using PEI MAX, harvested at 48 h after transfection, suspended in 300 µl of PBS, and underwent measurement of luciferase-derived luminescence intensity using Nano-Glo® Live Cell Substrate (Cat. No. N205A, Promega) and GloMax®20/20 Luminometer (Promega). In the case of CCCP treatment, Parkin-stable HeLa cells [66,81] co-transfected with pBiT 1.1 and pBiT 2.1 vectors were exposed to 20 µM CCCP (Wako Pure Chemical Industries, Ltd., Japan) or DMSO (as a negative control) for 4 h before NanoBiT assay and the intensities of the luciferase-derived luminescence were measured. To support the specificity of the intracellular interaction of BAG6 and UBL4A, this expression system used particularly weak promoters to ensure very low expression levels of the NanoBiT tag-fused proteins.

### Statistics

Statistical analyses were performed using a Welch's *t*-test, if not stated otherwise, and quantified data are presented as mean ± S.D., as indicated in the figure legends. All experiments involved at least three independent biological replicates to compute statistical significance. A *P*-value <0.05 was considered statistically significant.

### Data reproducibility

All individual experiments described have been repeated at least three times, and many have been repeated between three and six times. Initial observations of UBL4A dissociation from BAG6 by proteotoxic stresses were made by Dr. Ryosuke Minami. These were reproduced independently by Ms. Chizuru Ushio and Mr. Takumi Hagiwara and then expanded on, with NanoBiT analysis, by Mr. Takumi Hagiwara. Therefore, key results have been repeated independently by three experimentalists.

## Data Availability

All original data included in this paper are available from the authors upon request. Nucleotide sequence information of plasmids used in NanoBiT analysis were deposited to DDBJ DNA data bank of Japan (Accession number LC773210 and LC773211) [83]. This paper did not include datasets listed below: -Structural/crystallographic data for both macromolecular structures and small molecules -Functional genomics and molecular interactions/proteomics/metabolomics data -Computational models -Genetics data (genetic polymorphisms; genotype data).

## Abbreviations

BAG: BCL2-associated athanogene
BAGS: BAG-similar
CCCP: carbonyl cyanide m-chlorophenyl hydrazone
CHX: cycloheximide
DAPI: diamidino-2-phenylindole
DMSO: dimethyl sulfoxide
DRPLA: dentatorubral-pallidoluysian atrophy
ER: endoplasmic reticulum
ERAD: endoplasmic reticulum-associated degradation
GET: guided entry of TA proteins
GFP: green fluorescent protein
IP: immunoprecipitation
LgBiT: large-BiT tag
MG-132: benzoyl-leucyl-leucyl-leucyl-aldehyde
NS: not significant
PBS: phosphate-buffered saline
PolyQ: polyglutamine
Ppt.: pellet
Q32: polyQ tracts of normal (32) length residues
Q79: polyQ tracts of pathological (79) length residues
RLU: relative luminescence unit
SDS–PAGE: sodium dodecyl sulfate-polyacrylamide gel electrophoresis
SmBiT: small-BiT tag
SNARE: N-ethylmaleimide-sensitive factor attachment receptors
Sup.: supernatant
TA: tail-anchored
TMD: transmembrane domain
TRC: transmembrane domain recognition complex
TUGS: tethering UBL4A to BAG6
UBL: ubiquitin-like
