# Supplementary material for: Proteotoxic stresses stimulate dissociation of UBL4A from the tail-anchored protein recognition complex
Source: Biochem J. 2023 Oct 11;480(19):1583–98. doi: 10.1042/BCJ20230267 (PMC10586765; doi:10.1042/BCJ20230267)
Supplement: Supplementary Material [file BCJ-480-1583-s1.pdf]

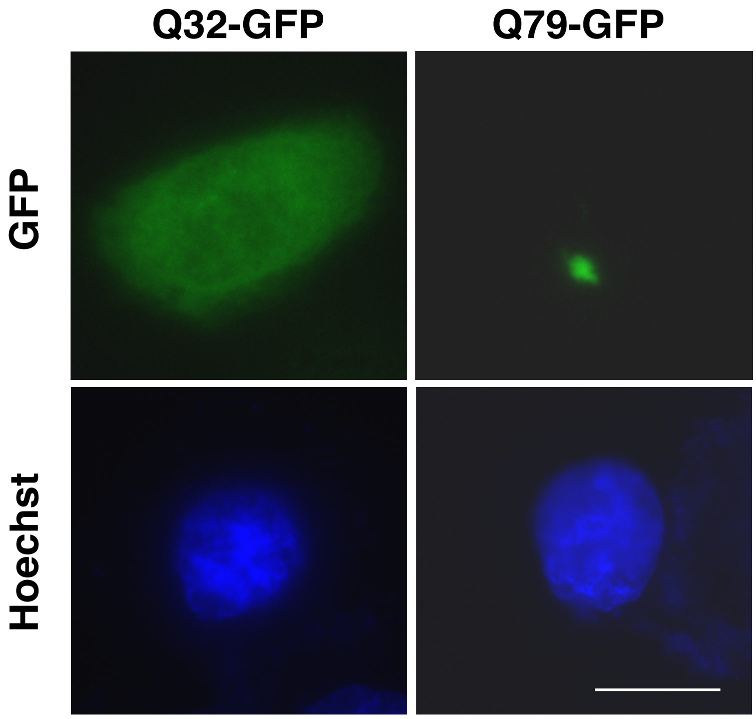

Supplemental Figure S1, Hagiwara et al.

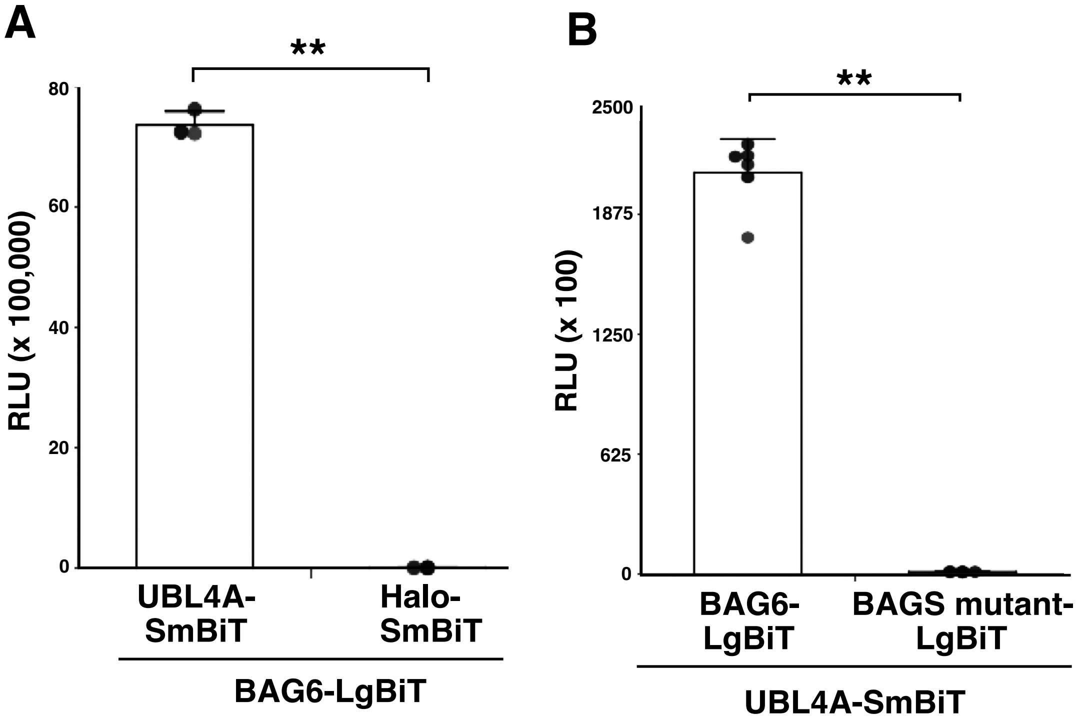

Supplemental Figure S2, Hagiwara et al.

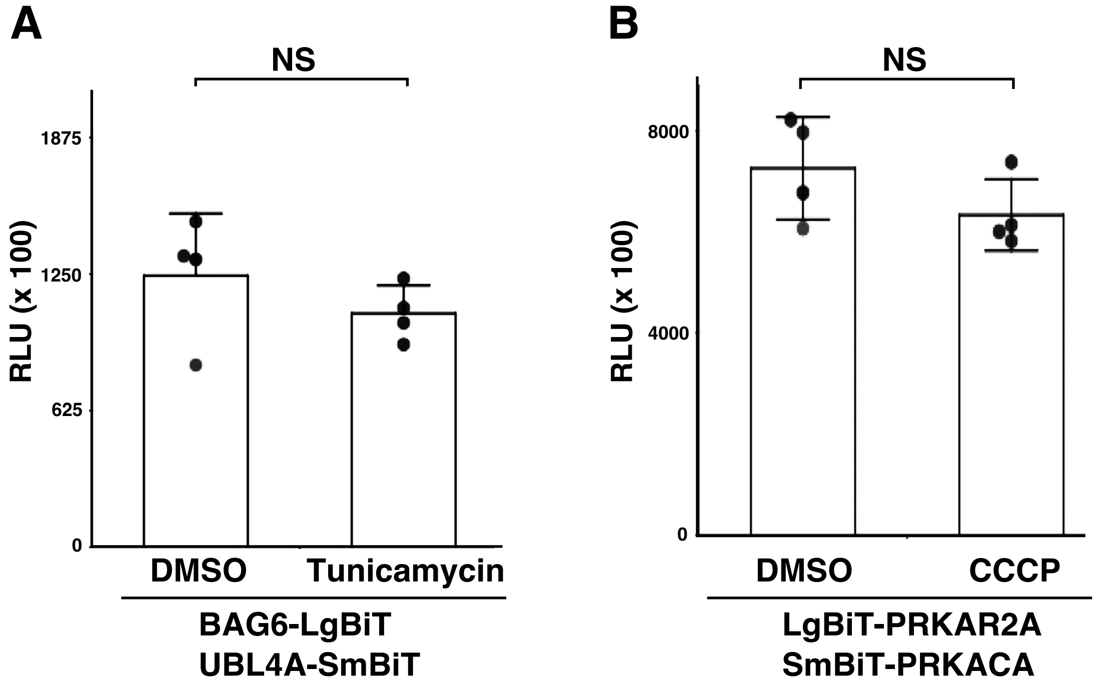

Supplemental Figure S3, Hagiwara et al.

SUPPLEMENTAL FIGURE LEGENDS

Figure S1, Related to Fig. 5, expression of long polyQ chain results in protein aggregate formation in HeLa cells.

A polyglutamine inclusion body was induced by the expression of atrophin-1-derived expanded polyQ protein in HeLa cells. HeLa cells were transfected with a Q79-GFP or Q32-GFP expression vector, then extracted with 1% Triton X-100. Fixed cells were stained with an anti-GFP antibody and Hoechst DNA stain. In the case of Q32-GFP expression, green signal was diffusely distributed throughout the cytosol and nucleus, while the expression of Q79-GFP induced inclusions. Green indicates the localization of polyQ-GFP proteins, and blue indicates Hoechst DNA stain. Scale bar: 10 μm.

Figure S2, Related to the experiments in Fig. 5E and Fig. 6, the intensity of the NanoBiT-derived luminescence signal reflects BAG6 binding to UBL4A in cells.

(A) NanoBiT-derived luminescence is dependent on the expression of UBL4A. Twenty-four hours after transfection of expression vectors encoding BAG6-LgBiT with UBL4A-SmBiT or Halo-SmBiT (negative control), NanoBiT-derived luminescence signals were measured, and the respective values normalized by cell number (RLU) were plotted on a graph. Welch's t test, n=3. **: p-value < 0.01.

(B) NanoBiT-derived luminescence is dependent on the interaction between UBL4A and BAG6. The hydrophobic residues Val^{1068} and Leu^{1086} of BAG6 in the Mock BAG/BAGS domain are critical for the interaction with UBL4A, and their point mutations to hydrophilic Arg residues have been reported to abolish binding to UBL4A [62]. NanoBiT-derived

luminescence (RLU) was also abolished with BAGS mutation of BAG6, supporting the conclusion that the luminescence observed in this assay reflects the binding of BAG6 and UBL4A in the cell. Welch's *t* test, n=6. **: *p*-value < 0.01.

Figure S3, Related to Fig. 6, mitochondrial depolarization leads to dissociation of the UBL4A-BAG6 complex.

(A) BAG6-UBL4A complex was not significantly affected by tunicamycin treatment. Twenty-four hours after transfection with BAG6-LgBiT and UBL4A-SmBiT expression vectors, HeLa cells were treated with 100 ng/mL tunicamycin for 4 h, and NanoBiT-derived luminescence (RLU) was measured. Treatment with dimethyl sulfoxide (DMSO) was used as a negative control. Welch's *t* test, n=4. NS; not significant.

(B) Although the NanoBiT system has been reported to not be affected by ATP concentration, we examined whether CCCP treatment (mitochondrial depolarization) affected the positive control signal derived from LgBiT-PRKAR2A and SmBiT-PRKACA. We confirmed that 20 μM CCCP treatment for 4 h did not significantly affect the luminescence signal intensity derived from PRKAR2A-PRKACA interaction. Welch's *t* test, n=4. NS: not significant.
